# Hepatitis B Blood Donor Screening Data: An Under-Recognized Resource for Canadian Public Health Surveillance

**DOI:** 10.3390/v15020409

**Published:** 2023-02-01

**Authors:** Sheila F. O’Brien, Cassandra N. Reedman, Carla Osiowy, Shelly Bolotin, Qi-Long Yi, Lillian Lourenço, Antoine Lewin, Mawuena Binka, Niamh Caffrey, Steven J. Drews

**Affiliations:** 1 Epidemiology and Surveillance, Canadian Blood Services, Ottawa, ON K1G 4J5, Canada; 2 School of Epidemiology and Public Health, University of Ottawa, Ottawa, ON K1N 6N5, Canada; 3 Public Health Agency of Canada, Ottawa, ON K1A 0K9, Canada; 4 National Microbiology Laboratory, Public Health Agency of Canada, Winnipeg, MB R3E 3P6, Canada; 5 Department of Medical Microbiology and Infectious Diseases, University of Manitoba, Winnipeg, MB R3T 2N2, Canada; 6 Department of Internal Medicine, University of Manitoba, Winnipeg, MB R3T 2N2, Canada; 7 Center for Vaccine Preventable Disease, University of Toronto, Toronto, ON M5S, Canada; 8 Dalla Lana School of Public Health, University of Toronto, Toronto, ON M5S, Canada; 9 Department of Laboratory Medicine and Pathobiology, University of Toronto, Toronto, ON M5S, Canada; 10 Public Health Ontario, Toronto, ON M5G 1V2, Canada; 11 Héma-Québec, Montreal, QC H4R 2W7, Canada; 12 Faculty of Medicine & Health Sciences, University of Sherbrooke, Sherbrooke, QC J1K 2R1, Canada; 13 BC Centre for Disease Control, Vancouver, BC V5Z 4R4, Canada; 14 School of Population and Public Health, University of British Columbia, Vancouver, BC V6T 1Z4, Canada; 15 Medical Microbiology Department, Canadian Blood Services, Edmonton, AB T6G 2R3, Canada; 16 Department of Laboratory Medicine & Pathology, Division of Diagnostic and Applied Microbiology, University of Alberta, Edmonton, AB T6G 2R3, Canada

**Keywords:** blood donors, chronic hepatitis B, vaccination

## Abstract

Hepatitis B surveillance is essential to achieving Canada’s goal of eliminating hepatitis B by 2030. Hepatitis B rates, association of infection with vaccine age-eligibility, and risk factors were analyzed among 1,401,603 first-time Canadian blood donors from 2005 to 2020. Donors were classified as having likely chronic or likely resolved/occult infections based on hepatitis B surface antigen, anti-hepatitis B core antigen, and hepatitis B nucleic acid test results. Likely chronically infected and control donors (ratio 1:4) participated in risk-factor interviews. The 2019 rate of likely chronic infection was 61.9 per 100,000 (95% CI 46.5–80.86) and 1449.5 per 100,000 for likely resolved/occult infections (95% CI 1370.7–1531.7). Likely chronic infections were higher in males (OR 3.2; 95% CI 2.7–3.7) and the vaccine-ineligible birth cohort (OR 1.9; 95% CI 1.6–2.2). The main risk factors were living with someone who had hepatitis (OR 12.5; 95% CI 5.2–30.0) and ethnic origin from a high-prevalence country (OR 8.4; 95% CI 5.9–11.9). Undiagnosed chronic hepatitis B may be more prevalent in Canada than currently determined by traditional passive hepatitis B reporting. Blood donor data can be useful in informing hepatitis B rates and evaluating vaccination programs in Canada.

## 1. Introduction

Transmissible by blood and other body fluids, the hepatitis B virus (HBV) is spread through sexual contact, sharing used injection equipment, invasive medical or dental procedures, as well as vertically, from mother to child [1]. About 90–95% of acute adult hepatitis B infections resolve spontaneously, but the remaining individuals develop chronic infections. Among children, up to 95% of infants, 50% of children under 5 years, and 10% of adolescents with acute hepatitis B go on to develop chronic hepatitis B [2]. Once chronically infected, there is no cure, but infection can be treated with lifelong antiviral medication [3,4]. Many people are unaware of their infection [5], and untreated hepatitis B infection can lead to cirrhosis and/or liver diseases [6]. 

Vaccination is more than 98% effective in preventing hepatitis B infection [7]. Both infant and pre-adolescent vaccination programs have reduced incidence globally [2,8]. All provinces and territories in Canada have instituted immunization programs since the early to mid-1990s (Table 1) [2,9]; however, gaps remain in Canadian vaccination coverage. Not all provinces have adopted a Canadian consensus conference recommendation for universal birth dose or infant vaccination [9,10]. Immigrants from higher hepatitis B prevalence countries (particularly in Africa and Asia) are more likely to experience chronic infections—even though vaccination is increasingly available—which can pose a risk to their Canadian-born children [8,11,12]. Hence, chronic hepatitis B infections may develop prior to infant or pre-adolescent vaccination or prior to immigrating.

Hepatitis B is a notifiable disease, similar to its status in the US, Europe, and elsewhere [13,14,15]. Surveillance involves routine annual reporting of hepatitis B diagnoses (with varying definitions regarding acute, chronic, or unspecified) from local public health professionals within provinces and territories to the Public Health Agency of Canada (PHAC), which then prepares national case counts and period prevalence [16,17]. These results are well known to underestimate the true chronic hepatitis B rate. Most people with chronic hepatitis B are asymptomatic, or have mild symptoms (e.g., fatigue), and may not exhibit known hepatitis B risk factors and hence, are not tested and are unaware of their infection. 

Canadian Blood Services (CBS) collects blood donations from nine out of ten Canadian provinces (not Quebec and the territories) in all large cities, plus many smaller urban centers and towns. With about 80,000 first-time donors annually, blood donors are largely an untapped resource for the public health surveillance of sexually transmitted and blood borne infection (STBBI). To protect transfusion recipients, blood services world-wide defer at-risk donors and test for hepatitis B and other disease markers [18]. Although not fully representative of the general population [19,20], blood donors can provide ongoing insight into the silent, asymptomatic, chronic hepatitis B population less likely to be identified by other sources. We have combined blood operator and public health expertise to examine whether blood donor hepatitis B testing data can be a continuous informative source for better understanding chronic hepatitis B prevalence in Canada. Our objectives are to examine the potential for blood donor data to contribute to hepatitis B public health surveillance through measuring chronic and likely resolved/occult hepatitis B (i) rates from 2005 to 2020 and their associated risk factors, and (ii) rates stratified by vaccine age eligibility (based on provincial infant and school-based vaccination programs).

## 2. Methods

### 2.1. CBS Donor Screening Questions

Prior to donating blood, CBS donors are required to answer screening questions to ensure they are in good health and are not at risk of blood transmissible infections. Table 2 describes reasons a donor may be deferred from donating blood based on screening questions that relate to sexual and percutaneous risk factors for hepatitis B infection, as well as health history. Prior to providing written consent to donate blood, donors are informed that their blood will be tested for a battery of infectious disease markers and other factors [21]. 

### 2.2. Testing Methodology 

Since 1972, all blood donations have been screened for hepatitis B surface antigen (HBsAg). As of 2005, all blood donations have been screened for HBsAg using the Abbott PRISM HBsAg immunoassay (Abbott Diagnostics Division, Wiesbaden, Germany) and for total antibodies to hepatitis B core antigen (anti-HBc) using the Abbott PRISM HBcore assay (Abbott Diagnostics Division, Wiesbaden, Germany). Beginning in 2011, donations were tested using the HBV nucleic acid test (HBV NAT) with the Roche Cobas MPX nucleic acid test (Roche Diagnostics International Ltd., Rotkreuz, Switzerland) in pools of six. The limit of detection of the assay is 1.4 (1.2–1.7) IU/mL [22]. Prior to this, supplemental HBV NAT was carried out on anti-HBc reactive samples using the Roche Cobas Ampliscreen nucleic acid test (Roche Diagnostics International Ltd., Rotkreuz, Switzerland). HBsAg was confirmed by neutralization; there was no confirmatory assay for anti-HBc. For HBsAg and anti-HBC, if the initial test was reactive, two repeat tests were conducted, and the result were interpreted as reactive if two of three replicates were repeatedly reactive. For NAT, the first test was in a 6 unit minipool, and if reactive, each sample in the pool was tested individually. Donors are informed of a positive result by letter advising them to consult a physician, and they are indefinitely deferred. Public health authorities are notified of all positive tests, as required by law, and public health then follows up with the donor to advise them about the need for medical care.

### 2.3. Infection Definitions

Donors were considered likely to have resolved hepatitis B infection if reactive for anti-HBc, but no other markers (clinical scenario 1); likely to have chronic hepatitis B infection if HBsAg and anti-HBc reactive (may be HBV NAT reactive or not) (clinical scenario 2); and to have an occult infection if anti-HBc and HBV NAT reactive (clinical scenario 3) (See Table 3). When chronic HBV infection is assumed, we note that acute hepatitis B infection cannot be ruled out with single HBsAg and anti-HBc reactive results, as anti-HBc IgM was not tested.

### 2.4. Epidemiology Donor Database 

The Epidemiology Donor Database is maintained with SAS software (SAS Institute Inc., Cary, NC, USA) and contains donation, test results, and demographic data such as age, sex, and residential location for all Canadian blood donors (excluding Quebec and all Canadian territories [Northwest Territories, Yukon and Nunavut]). Data for this analysis includes hepatitis B test results, donation date, province of residence at the time of the first donation, sex, and age. The variable ‘province of residence’ was analyzed as recorded for donors residing in British Columbia, Alberta, Saskatchewan, Manitoba, or Ontario; however, a new variable was created called ‘the Atlantic region,’ which combined data from New Brunswick, Nova Scotia, Prince Edward Island, and Newfoundland and Labrador, due to geographic proximity and low positive test counts. Country of birth and ethnicity are not available. 

### 2.5. Case-Control 

All first-time donors with likely chronic hepatitis B infection test profiles (likely clinical scenario 2, Table 3) from 2005 to 2020 were invited to participate in a telephone interview concerning potential risk factors. Risk factors included percutaneous exposure, such as needlestick injuries, tattoo, piercing, acupuncture, and intravenous drug use; potential blood contact, such as intranasal drug use and medical exposure (transfusion); sexual risks, such as multiple partners and transactional sex; and country of birth and ethnicity. A 1:4 case: control ratio was used in this study. All hepatitis B positive donors received a standard notification letter informing them of their test results, advising them to seek medical attention, and informing them that they were permanently deferred from blood donation. Donors were then sent a letter inviting them to participate in a telephone interview. Once a likely clinical scenario 2 donor had completed an interview, control donors (i.e., those who had tested negative for HBsAg, NAT, anti-HBc and all other markers), matched by case age (+/−5 years), sex, first-time donation status, and geographic region, were randomly selected and invited to participate in the same way. If a control donor refused to participate or could not be contacted, another was selected until four control donors had been interviewed per likely clinical scenario 2 donor. The telephone interview used a scripted questionnaire which asked about known and potential risk factors and demographic factors. Of the 818 likely clinical scenario 2 donors, 249 (30.4%) participated, and of the 2811 controls invited, 996 (35.4%) participated. 

### 2.6. Statistical Analysis

#### 2.6.1. Comparison of First-Time Blood Donors with the General Population

The percentage of first-time donors in the study period was calculated for sex, age group, and region. Census data were used to obtain the corresponding percentages for people in the general population over the age of 17 (thus age-eligible to donate blood) and residing in the nine provinces where CBS collects blood donations [23].

#### 2.6.2. First-Time Donor Hepatitis B Period Prevalence and Factors Associated with Testing Positive for Hepatitis B Seromarkers

To describe donors by test results, donors were sorted into the two periods (9 April 2005 to 28 February 2011) according to when HBV NAT was a supplemental test, and (1 March 2011 to 31 December 2020) when HBV NAT was performed on each sample. Donors were then further classified by test results, the total tallied, and the percentage of all donors in the period calculated. 

The rates of hepatitis B-positive donors by likely clinical scenario data were calculated per 100,000 donors, broken down by demographic variables (sex, age group, and region), and 95% CI was estimated using the Clopper–Pearson Exact method.

Table 1 describes the first birth year when each province began implementing a routine hepatitis B vaccination program [9]. A dummy variable for the vaccine cohort was constructed, in which 1 = born in a vaccine eligible year (or vaccine eligible, i.e., born on or after the first birth year with routine vaccination), and 0 = born in a vaccine ineligible year (or vaccine ineligible, i.e., born prior to the first birth year with universal vaccination through an infant or school-based program). 

The number and rate per hundred thousand donors for clinical scenario 2 (likely chronic hepatitis B infection) and clinical scenarios 1/3 (likely resolved/occult infections) were calculated for each year for all donors and for the vaccine eligible and ineligible cohorts. Univariable logistic regression models were constructed with clinical scenario 2, and separately with clinical scenarios 1 and 3 as the dependent variable and sex, age, vaccine cohort, region, and year of donation as independent variables. Multivariable logistic regression models were conducted using significant (*p* < 0.05) univariate analysis variables. 

#### 2.6.3. Case-Control Study Analysis

Univariable logistic regression models were constructed for risk factors requested in the interview. Variables that were significant (*p* < 0.05) in the univariate analysis were included in a multivariable logistic regression model. Collinearity was assessed. Based on questions from the interview, two new variables were constructed to assess as potential risk factors: born in a hepatitis B highly endemic country (high-risk country of birth) and self-reported ancestral ethnic origin from a hepatitis B highly endemic country (high-risk ethnic origin). A country where hepatitis B prevalence was 5% or higher was considered to be high-risk [8]. A dummy variable was constructed where 1 = born in a high-risk country, and 0 = not born in a high-risk country. Ethnic origin was classified as high-risk if the donor self-reported their ancestral ethnicity to be from a high-risk country (5% or higher hepatitis B prevalence [8]); these ethnicities included Asian (unspecified, East, Southeast, West, and South), African, Chinese, and Filipino. If donors chose more than one ethnicity that would best describe their origin, if any of the ethnicities that they reported were considered high-risk (as previously described), they were treated as being of a high-risk ethnic origin. A dummy variable was constructed where 1 = high-risk ethnicity, and 0 = low-risk ethnicity.

## 3. Results

Between 9 April 2005, and 31 December 2020, there were 1,401,603 first-time blood donors in Canada. Table 4 shows similar percentages of males and females and in regions compared with the general population; first-time donors tend to be younger. Table 5 shows the breakdown by test results from 9 April 2005 to 28 February 2011, when hepatitis B NAT was a supplemental assay (usually performed if positive for anti-HBc) and from 1 March 2011 to 31 December 2020, when HBV NAT was performed on all donors. Across all study years, the most common test profile among donors who tested positive was clinical scenario 1 (likely resolved infections, 18,360 donors, 1.3%) followed by clinical scenario 2 (likely chronic infections, 787 donors, 0.087%). There were 79 anti-HBc reactive/HBsAg positive/NAT non-reactive donors, comprising about 10% of likely chronic infections. Donors associated with clinical scenario 2 comprised 0.06% of all donors. There were 61 donors classified as exhibiting clinical scenario 3 (likely occult infections) (0.004%). The rates of clinical scenarios 2 and 1/3 are shown by year in Supplemental Table S1. Supplemental Table S2 shows the 2019 breakdown of rates of clinical scenarios 2 and 1/3 in donors by sex, age, and region. The calculated hepatitis B-positive rate among first-time donors in clinical scenario 2 (likely chronic infection) was 61.9 per 100,000 (95% CI 46.5–80.8) and was over 20 times higher in clinical scenario 1/3 (1449.5 per 100,000 (95% CI 1370.7–1531.7)). Importantly, nearly all HBV-positive donations were anti-HBc reactive.

**Figure 1:**
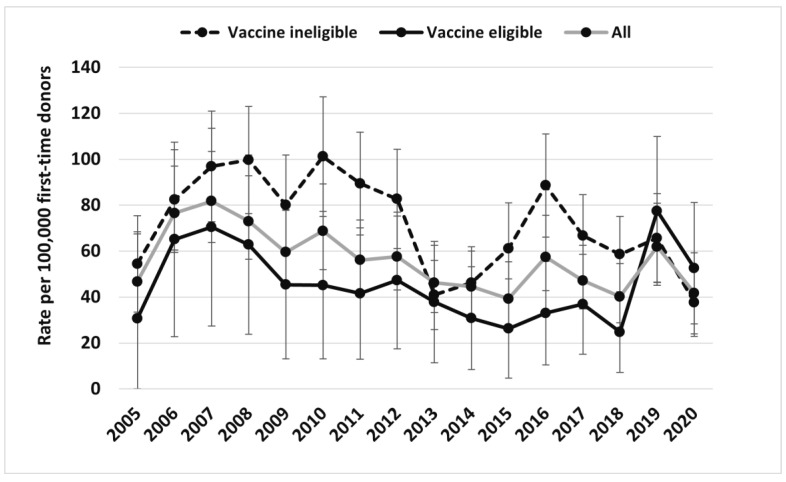
HBV clinical scenario 2 (likely chronic infection) rate (per 100,000 first-time donors) (95% confidence intervals) by vaccine age eligibility based on provincial routine programs from 2005–2020.

**Figure 2:**
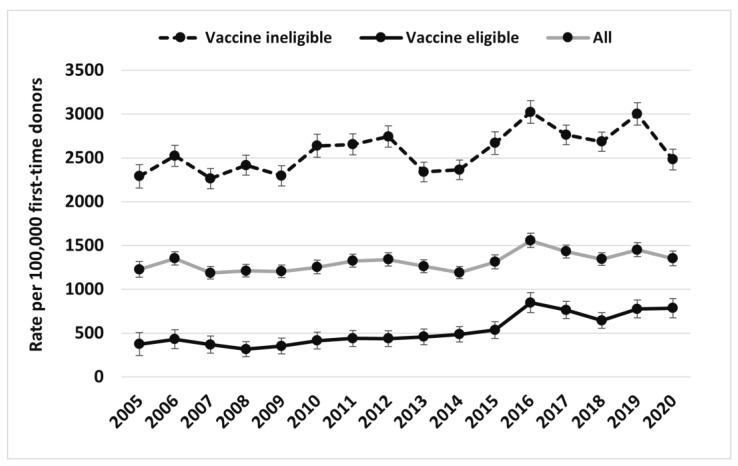
HBV clinical scenarios 1/3 (likely resolved/occult infection) rate (per 100,000 first-time donors) (95% confidence intervals) by vaccine age eligibility based on provincial routine programs from 2005–2020.

Figure 1 and Figure 2 illustrate overall trends of clinical scenarios 2 and 1/3 broken down by the vaccine age-eligible and age-ineligible cohorts from 2005 to 2020, respectively, as well as the rates for all donors combined. The vaccine age-eligible cohort includes those individuals who were in an age group that would have been offered vaccine (mean age 21.9 years old vs. 43.7 years old in the vaccine ineligible age cohort, *p* < 0.001). Table 6 shows the final multivariable regression model, with clinical scenario 2 as the dependent variable. The odds of clinical scenario 2 were higher in males than females (OR 3.2; 95% CI 2.7–3.7, *p* < 0.01), in those born in vaccine ineligible years (versus those not born in vaccine eligible years) (OR 1.9; 95% CI 1.6–2.2, *p* < 0.01), and in those residing in British Columbia compared with Ontario, but much lower in Manitoba and Atlantic Canada. Table 7 shows the final multivariable logistic regression model for donors with clinical scenarios 1/3 as the dependent variable. The odds of clinical scenario 1/3 were higher in males compared with females (OR 1.6; 95% CI 1.6–1.7, *p* < 0.01), in those born in vaccine ineligible years compared with those not born in vaccine eligible years (OR 3.5; 95% CI 3.3–3.7, *p* < 0.01), and in those living in British Columbia compared with Ontario, but much lower in Saskatchewan, Manitoba, and Atlantic Canada.

Risk factors identified in the univariate analysis are shown in Supplemental Table S3. In the multivariable logistic regression model, shown in Table 8, three independent risk factors were identified: a history of living with someone who had hepatitis (OR 12.5; 95% CI = 5.2–30.0), a history of ever receiving a blood transfusion (OR 3.3; 95% CI = 1.7–6.1), and ethnic origin from a region with a high hepatitis B prevalence (OR 8.4; 95% CI = 5.9–11.9). Having been born in a high prevalence country, rather than being of ethnic origin from a high prevalence region, was also a significant predictor (*p* < 0.001), but as these two variables were correlated (r = 0.47, *p* < 0.0001), it was not retained in the multivariable model. High-risk ethnic origin was chosen over high-risk country of birth due to better model fit and tighter 95% confidence intervals. For those donors who had the date of their transfusion recorded, 44.4% occurred after 1990. Of donors included in the case control analysis, 141 (56.6%) cases and 143 (14.4%) controls had at least one risk factor in the final model. Of donors with clinical scenario 2 in the case control analysis, 119 (47.8%) were from higher prevalence countries and 148 (59.4%) were from an ethnic background associated with higher prevalence countries. When donors with clinical scenario 2 were asked what their reaction was to their notification letter, every donor said they were surprised, most often shocked.

## 4. Discussion

Blood donors represent a healthy subset of the population unaware of their hepatitis B status at the time that are screened for recent risk factors, are in good general health and believe their blood is safe at the time of donation. With about 1.4 million donors tested over 15 years in 9 of 10 provinces, this is the largest study of its kind in Canada. This is the first report of Canadian blood donor hepatitis B infection status in relation to routine vaccination program age eligibility.

Most hepatitis B surveillance data in Canada and elsewhere are derived from the mandatory reporting of diagnosed community cases [13,14,15], which underestimate the true chronic hepatitis B rate [24]. Our 2019 ‘likely chronic’ hepatitis B rate of 61.9 per 100,000 (or 0.062%) is six times higher than the 2019 nationally reported rate (10 cases per 100,000) [17]. The Canadian Health Measures Survey (a randomized population study) reported an even higher rate than identified in blood donors—400 per 100,000 population (or 0.4%), compared with 67.6 per 100,000 (or 0.067%) for the 2007–2011 first-time blood donors [25]. However, the Canadian Health Measures Survey did not distinguish chronic from acute infections, nearly half of participants had a hepatitis B diagnosis (thus ineligible to donate blood), the study may contain a different mix of immigrants/ethnic groups, and it included more older people than first-time blood donors. While the public health definition relies on clinical history in addition to seromarkers (thus being be more specific), such data are biased towards people who access healthcare and have a reason to be tested. These include individuals with symptoms, high-risk contacts, or known risk factors, as well as those undergoing routine screening programs, such as in pregnancy [26,27]. However, most chronic hepatitis B cases are asymptomatic or have mild symptoms.

Our results follow similar chronic hepatitis B trends by sex, age, and province/region in the general population [17], with the highest rates among donors who are male, 30–39 years of age, and residing in BC, a province with higher proportions of people of Asian ancestry [17,28,29,30]. The increasing trend in resolved/occult infections in vaccine age eligible donations may be related to changing demographics, with more immigrants from higher prevalence countries as the cohort ages. In our case-control analysis, living with someone who had hepatitis, as well as ethnicity and/or birth in a hepatitis B high prevalence country were associated with likely chronic hepatitis B. However, nearly half of the likely chronically infected donors did not have any of these risk factors and were thus less likely to be tested and diagnosed by Canadian hepatitis B screening recommendations [12]. The association of blood transfusion with chronic hepatitis B is interesting and may be related to transfusion in other countries, or function as a proxy for nosocomial infections, as many occurred prior to improvements in hospital infection control practices. Transfusion-transmitted hepatitis B has not been reported in Canada for over 20 years. Our results suggest that there is a substantial segment of the population with chronic hepatitis B not being identified by the healthcare system, nor followed to determine if treatment is required. The pool of potentially infectious individuals unaware of their risk of transmitting hepatitis B to others is also substantial [24].

Blood donor surveillance data can lend valuable insight into hepatitis B prevalence in the asymptomatic Canadian population and should be considered in tandem with public health surveillance data. Blood services have a variety of structures and relationships with public health. In England and France, blood donor surveillance data is under the umbrella of public health. In the US and many European countries, blood collection is carried out by independent regional blood centers [31]. Regional data can contribute nationally, such as via the US Transfusion Transmissible Infections Monitoring System, which collates data from blood centers to monitor blood safety and could be an adjunct to public health surveillance [32].

The World Health Organization has set an ambitious target for eliminating hepatitis B by 2030 [33]. Surveillance and strategies to identify and treat infected individuals are fundamental to this aim, but vaccination prior to exposure is paramount. In our study, older individuals could have other risks. Those born in higher prevalence countries are more likely to have hepatitis B [3,12,34], although in our case control analysis, nearly half of donors with likely chronic infection were born in Canada. That both chronic and resolved hepatitis B infections are less common in vaccine eligible age cohorts suggests that childhood vaccination is impactful. Nevertheless, infections were identified in the vaccine age-eligible cohort. Less than optimal hepatitis B prevention may be related to imperfect vaccination coverage (currently about 75%) and pre-natal prevention strategies [35,36,37,38]. 

Although in 2009, the WHO recommended that low-endemic countries, such as Canada, provide universal birth dose vaccinations [39], as of 2020, only 3 out of 13 provinces/territories have birth dose vaccinations, 5 start at 2 months, and 5 vaccinate in early adolescence [9]. Adolescent vaccination strategies aim to prevent infection prior to sexual debut and are predicated on the assumption of effective pregnancy screening and post-exposure prophylaxis for babies, where appropriate. In Ontario, where vaccination is offered in early adolescence, only 92.7% of pregnancies were screened for HBsAg, and follow-up was sub-optimal [27]. Thus, although the vaccine is very effective, the combination of immigrants infected overseas and Canadian vaccination programs that have imperfect uptake and are not always offered to prevent early childhood infection are likely factors behind the observed infections in our vaccine age-eligible blood donors.

The evaluation of hepatitis B vaccination programs is achieved via vaccination records and public health reported infections, supplemented by studies and evaluations of specific programs. In Canada, randomized surveys and provincial vaccination records assess vaccine coverage among children, but there is no national repository of vaccination records, and provincial records do not cover the lifespan of most adults [35,36]. Blood donor data has been applied to evaluate vaccination programs in South Africa, Belgium, and China, but focused on the blood safety benefit [40,41,42]. We believe that our analysis shows that blood donor data are useful as a component of vaccination evaluation, ideally used collaboratively with provincial and national data.

We defined likely chronic infections as those that showed up in donors who were both anti-HBc reactive, using an Ig total assay, and HBsAg positive, confirmed by a neutralization test. Most were also HBV NAT reactive. However, an acute infection may be anti-HBc IgM positive, which our assay did not distinguish from IgG. Hence, although all donors were symptom free and did not exhibit recent risk factors, some acute infections could have been included [43]. Likely occult infections were defined as HBsAg negative, with both anti-HBc reactive and HBV NAT reactive results [43]. We assumed that anti-HBc reactivity, when neither HBsAg positive nor HBV NAT were reactive, signified resolved infections, although some occult infections are possible, as nucleic acid concentrations can be below detection [44,45]. For this reason, and because occult infection may be less likely to predict long term sequalae [46], we analyzed occult and resolved infections together.

Important limitations of our study are that viral concentrations were not measured, and vaccination, symptom history, and country of birth were not available in the surveillance data. As recruitment practices target younger people (under 30 years), they are over-represented in the sample, but as chronic hepatitis B is more likely to be asymptomatic in younger people, they may be less frequently captured in public health case reports. Key strengths of this study include the Canada-wide sample collection and testing of all donors. An interesting feature of our study is the analysis of resolved and occult infections. While Canadian reported case counts of acute and chronic infections are available [17], data on resolved hepatitis B infections and occult infections are scant because these are not reported from surveillance systems. In a publication of the Canadian Health Measures Survey, 3.3% of their participants had resolved infections, which included people who were aware they had hepatitis [25]. In our study 1.3% of donors unaware of their infection history had resolved infections (this includes a small number of occult infections).

## 5. Conclusions

In Canada, some World Health Organization hepatitis B elimination recommendations have been met, such as preparing treatment guidelines and blood safety measures. Conversely, not all people with hepatitis B receive recommended follow-up testing [47,48], follow-up care is inconsistent [12], and while pharmacologic therapy is available, the specific treatment options vary across jurisdictions [49]. Our surveillance results suggest that undiagnosed chronic hepatitis B may be more common in Canada than is suggested by national statistics, and that blood donor data can be useful in evaluating vaccination programs. While we focus on the Canadian situation, leveraging the intersection between blood donor and public health surveillance data could enhance the laboratory and epidemiological surveillance of bloodborne infections in many countries.

## Figures and Tables

**Table 1:** Calendar years when free routine hepatitis B programs have been offered, and the first birth year with routine hepatitis B vaccination programs by province in Canada.

Province	Type of Hepatitis B Vaccination Program		First Birth Year Eligible to Receive Free Routine Hepatitis B Vaccination
	Routine Infant Program	Routine School-Based Program ^1^	
British Columbia ^6^	2001–present	1992–2001 (6th grade)	1981
Alberta ^6^	2018–present	1995–present (5th grade)	1985
Saskatchewan	-	1995–present (6th grade)	1984
Manitoba	-	1998–2017 (4th grade) ^2^	1989
Ontario	-	1994–present (7th grade)	1982
New Brunswick ^5^	1995–present	1995–2005 (4th grade)	1986
Prince Edward Island ^6^	1995–present	1995–2010 (3rd grade)	1987
Nova Scotia	-	1995–2010 (4th grade) ^3^	1986
Newfoundland	-	1995–2012 (4th grade) ^4^	1986

^1^ Public Health Agency of Canada (PHAC), 2021. Hepatitis B vaccine: Canadian Immunization Guide, available from: https://www.canada.ca/en/public-health/services/publications/healthy-living/canadian-immunization-guide-part-4-active-vaccines/page-7-hepatitis-b-vaccine.html, accessed on 18 March 2022; ^2^ grade 4 program moved to grade 6 in 2017; ^3^ grade 4 program moved to grade 7 in 2010; ^4^ grade 4 program moved to grade 6 in 2012; ^5^ first dose at birth; ^6^ first dose at 2 months.

**Table 2:** Reasons a donor may be deferred from donating blood based on pre-donation screening questions related to HBV sexual or percutaneous risk factors.

Reasons for Donation Deferral
Feeling unwell
Post-exposure hepatitis B prophylaxis
Had hepatitis B or C, ever
Had hepatitis (other than B or C) in last 6 months
Exposure to someone with hepatitis (viral or cause unknown) in last 12 months
Tattoo, skin, or ear piercing in last 3 months
Acupuncture or electrolysis in last 6 months (if not single use needle)
Needlestick injury in last 6 months
Intravenous drug use, ever
Sex with a sex trade worker in last 12 months
Received money or drugs for sex since 1977
Lived in certain Sub-Saharan African countries since 1977 (discontinued in 2018)

Note: The time frame for some deferral reasons have varied over the reporting period (2005 to 2020).

**Table 3:** Hepatitis B test profiles and likely clinical scenarios.

Test Profile	‘Likely Clinical Scenario’ Number and Name
1. Anti-HBc reactive HBsAg negative HBV NAT negative	1. Resolved infection
2. Anti-HBc reactive HBsAg positive HBV NAT positive or negative	2. Chronic infection *
3. Anti-HBc reactive HBsAg negative HBV NAT positive	3. Occult infection
4. Anti-HBc non-reactive HBsAg negative HBV NAT negative	4. Never infected

* includes all potential phases of chronic infection (HBeAg positive or negative chronic infection or hepatitis).

**Table 4:** Breakdown of the percentage of first-time blood donors (April 2005 to December 2020) by sex, age group, and region compared to the age-eligible Canadian population.

Variable	Categories	First-Time Blood Donors (%)	Canadian General Population (17 Years and Older) ^a^ (%)
Sex	Female	54.0	51.3
	Male	46.0	48.7
Age group	17–29	53.5	19.7
	30–39	17.2	17.0
	40–49	14.7	32.2
	50+	14.6	31.1
Region	British Columbia	15.2	18.1
	Alberta	18.1	14.5
	Saskatchewan	4.8	3.7
	Manitoba	5.9	4.5
	Ontario	46.8	50.3
	Atlantic Canada	9.2	8.7

^a^ Statistics Canada 2021 Census data, Census Profile, 2021 Census of Population. Limited to population 17 years and older to reflect the population eligible to donate blood and excluding the province and territories where CBS do not collect donations (Québec, Northwest territories, Yukon, and Nunavut). Atlantic region includes Newfoundland and Labrador, Prince Edward Island, Nova Scotia, New Brunswick. From table 98100026-eng.zip. Available from: https://www150.statcan.gc.ca/n1/tbl/csv/98100026-eng.zip (accessed on 31 January 2023).

**Table 5:** Breakdown of hepatitis B marker (anti-HBc, HBsAg, HBV NAT) test results among first-time donors (2005–2020).

	9 April 2005 to 28 February 2011	1 March 2011 to 31 December 2020	Total
Number of first-time CBS donors	507,165	894,438	1,401,603
Type of hepatitis B screening test performed on first-time CBS donors	Overall number of hepatitis B screening tests performed on first-time CBS donors (% positive)		
Anti-HBc	507,165 (1.31)	894,438 (1.41)	1,401,603 (1.37%)
HBsAg	507,165 (0.07)	894,438 (0.05)	1,401,603 (0.056%)
NAT ^1^	6681 (5.03)	894,438 (0.05)	See footnote 1
Hepatitis B screening test	Number of first-time CBS donors testing positive (Percent of positive tests %)		
Anti-HBc only ^2^	6252 (1.23)	12,108 (1.35)	18,360 (1.31%)
NAT only ^3^	-----	2 (0.0002)	See footnote 1
HBsAg only ^4,5^	5 (0.001)	4 (0.0006)	9 (0.0006%)
Anti-HBc and HBsAg only ^4^	40 (0.008)	39 (0.004)	79 (0.006%)
Anti-HBc and NAT only ^2^	33 (0.007)	28 (0.003)	61 (0.004%)
HBsAg and NAT only ^4,5^	1 (0.01)	1 (0.0001)	2 (0.0001%)
Anti-HBc and HBsAg and NAT ^4^	302 (0.06)	395 (0.04)	697 (0.05%)
All positive anti-HBc tests	6627 (1.31%)	12,570 (1.41%)	19,197 (1.37%)
All positive HBsAg tests	348 (0.07%)	439 (0.05%)	787 (0.056%)
All positive NAT ^1^ tests	See footnote 1	426 (0.05%)	See footnote 1

^1^ NAT testing was supplementary prior to 28 February 2011; ^2^ likely occult/resolved infections (clinical scenarios 1,3); ^3^ likely incident infections; ^4^ likely chronic infections (clinical scenario 2); ^5^ potentially false positive (occurs when original signal is near cut-off with “false” neutralization). Note: Seven samples that were HBsAg repeat reactive did not complete confirmatory testing. However, they were repeat reactive for anti-HBc and therefore, were considered HBsAg positive and classified as likely chronic infections.

**Table 6:** Output from multivariable logistic regression model, with clinical scenario 2 (likely chronic infection) as the dependent variable.

Variable	Categories	Odds Ratio (OR)	95% CI
Year of donation	-	0.972	0.957–0.988
Sex	Female	Ref. ^1^	-
	Male	3.150	2.689–3.691
Birth Cohort	Hepatitis B vaccine eligible	Ref. ^1^	-
	Hepatitis B vaccine ineligible	1.871	1.616–2.166
Province or region of residence *	Ontario	Ref. ^1^	-
	British Columbia	1.256	1.043–1.512
	Alberta	0.939	0.777–1.135
	Saskatchewan	0.897	0.635–1.268
	Manitoba	0.667	0.475–0.935
	Atlantic Canada **	0.219	0.136–0.351

^1^ Ref. = Referent category in the model. * Province or region of residence at the time of hepatitis B first-time blood donor screening. ** Atlantic Canada is a summary of first-time donors who were screened for hepatitis B who resided in the provinces of New Brunswick, Nova Scotia, Prince Edward Island, and Newfoundland and Labrador at the time of blood donor screening.

**Table 7:** Output from multivariable logistic regression model, with clinical scenarios 1/3 (likely resolved/occult infection) as the dependent variable.

Variable	Categories	Odds Ratio (OR)	95% CI
Year of donation	-	1.013	1.010–1.017
Sex	Female	Ref. ^1^	-
	Male	1.641	1.593–1.691
Age	-	1.020	1.018–1.021
Birth Cohort	Hepatitis B vaccine eligible	Ref. ^1^	-
	Hepatitis B vaccine ineligible	3.513	3.328–3.708
Province or region of residence at the time of blood donation	Ontario	Ref. ^1^	-
	British Columbia	1.240	1.193–1.289
	Alberta	0.973	0.936–1.012
	Saskatchewan	0.626	0.574–0.682
	Manitoba	0.699	0.652–0.750
	Atlantic Canada	0.284	0.261–0.309

^1^ Ref. = Reference category in the model. Sensitivity analysis: considering the seven HbsAg that were repeat reactive, but not confirmed positive/Anti-HBc positive samples, as resolved/occult infections instead of chronic infections made only minor changes to any parameters within either model.

**Table 8:** Output from the multivariable logistic regression model of case-control interviews, with clinical scenario 2 (likely chronic infection) as the dependent variable *.

Variable	Odds Ratio (OR)	95% CI	*p* Value
Received a blood transfusion (ever)	3.26	1.74–6.11	0.0002
Lived with someone who had hepatitis (any)	12.46	5.18–29.97	<0.0001
High-risk ethnic origin ^1^	8.39	5.91–11.91	<0.0001

^1^ Ethnic origins from highly endemic HBV countries (>5% prevalence). People of African, Asian (East, Southeast, West, and South, unspecified), Chinese, and Filipino ethnic origin have an increased likelihood of having been born in or having lived with people born in HBV-endemic countries and were considered “high-risk”. * Results from univariate analysis are available in Table S3.

## Data Availability

Summary data are available on request from sheila.obrien@blood.ca.

## Supplementary Materials

The following supporting information can be downloaded at: https://www.mdpi.com/article/10.3390/v15020409/s1, Table S1. Hepatitis B annual positive rate (per 100,000) among first-time CBS donors, 2005 to 2020. Table S2. Breakdown of HBV-positive rates by clinical scenario among first-time donors by sex, age, and region, 2019. Table S3. Output from a univariate logistic regression model of case-control interviews, with clinical scenario 2 (likely chronic infection) as the dependent variable.
