# Supplementary material for: Hepatitis B Blood Donor Screening Data: An Under-Recognized Resource for Canadian Public Health Surveillance
Source: Viruses. 2023 Feb 1;15(2):409. doi: 10.3390/v15020409 (PMC9966614; doi:10.3390/v15020409)
Supplement: Supplementary file 1 [file viruses-15-00409-s001.zip › viruses-2175738-supplementary.pdf]

Supplementary Materials:

Table S1. Hepatitis B annual positive rate (per 100,000) among first-time CBS donors, 2005 to 2020

Year	Total n	Clinical Scenario 2 Likely Chronic hepatitis B		Clinical Scenarios 1/3 Likely Resolved/Occult hepatitis B	
		Positive n	Rate per 100,000 (95% CI)	Positive n	Rate per 100,000 (95% CI)
**2005**	59,979	28	46.7 (31.0 - 67.5)	735	1,225.4 (1138.4 - 1317.3)
**2006**	88,804	68	76.6 (59.5 - 97.1)	1,200	1,351.3 (1275.9 - 1430.0)
**2007**	85,591	70	81.8 (63.8 - 103.3)	1,016	1,187.0 (1115.2 - 1262.3)
**2008**	90,490	66	72.9 (56.4 - 92.8)	1,095	1,210.1 (1139.5 - 1283.9)
**2009**	87,389	52	59.5 (44.4 - 78.0)	1,052	1,203.8 (1132.2 - 1278.8)
**2010**	81,471	56	68.7 (51.9 - 89.3)	1,020	1,252.0 (1176.3 - 1331.2)
**2011**	92,745	52	56.1 (41.9 - 73.5)	1,228	1,324.1 (1251.0 - 1400.3)
**2012**	92,066	53	57.6 (43.1 - 75.3)	1,234	1,340.3 (1266.6 - 1417.3)
**2013**	90,886	42	46.2 (33.3 - 62.5)	1,147	1,262.0 (1190.0 - 1337.2)
**2014**	96,543	43	44.5 (32.2 - 60.0)	1,148	1,189.1 (1121.3 - 1259.9)
**2015**	81,589	32	39.2 (26.8 - 55.4)	1,070	1,311.5 (1234.1 - 1392.5)
**2016**	88,806	51	57.4 (42.8 - 75.5)	1,382	1,556.2 (1475.2 - 1640.5)
**2017**	101,755	48	47.2 (34.8 - 62.6)	1,456	1,430.9 (1358.3 - 1506.3)
**2018**	102,037	41	40.2 (28.8 - 54.5)	1,371	1,343.6 (1273.5 - 1416.7)
**2019**	87,201	54	61.9 (46.5 - 80.8)	1,264	1,449.5 (1370.7 - 1531.7)
**2020**	74,251	31	41.8 (28.4 - 59.3)	1,003	1,350.8 (1268.5 - 1437.1)
**Overall 2005 to 2020**	1,401,603	787	56.1 (52.3-62.1)	18,421	1314.3 (1295.5 – 1333.4)

Table S2. Breakdown of HBV-positive rates by clinical scenario among first-time donors by sex, age, and region, 2019.

Demographics	Clinical Scenario 2 Likely Chronic		Clinical Scenario 1 and 3 Likely Occult and Resolved	
	Total Tested	Rate/100,000 first-time donors (95% CI)	Total Tested	Rate/100,000 first-time donors (95% CI)
**Sex**				
Female	46,117	36.9 (21.5 - 59.0)	46,117	1,047.3 (956.0 - 1145.0)
Male	41,084	90.1 (63.4 - 124.1)	41,084	1,901.0 (1770.0 - 2039.1)
**Age Group**				
17-29	42,487	37.7 (21.5 - 61.2)	42,487	313.0 (262.1 - 371.0)
30-39	17,084	122.9 (76.1 - 188.0)	17,084	1,574.6 (1392.1 - 1774.5)
40-49	12,198	65.6 (28.4 - 129.3)	12,198	3,328.4 (3012.5 - 3668.5)
50+	15,432	58.3 (26.7 - 110.7)	15,432	2,954.9 (2689.9 - 3239.0)
**Province or Region of Residence***				
British Columbia	14,718	88.3 (47.1 - 151.1)	14,718	1,644.3 (1443.6 - 1865.0)
Alberta	16,873	35.6 (13.1 - 77.4)	16,873	1,535.0 (1353.8 - 1733.8)
Saskatchewan	3,554	140.7 (45.9 - 328.4)	3,554	1,012.9 (709.6 - 1402.4)
Manitoba	3,866	51.7 (6.5 - 187.0)	3,866	1,474.4 (1116.9 - 1910.5)
Ontario	40,789	63.7 (41.7 - 93.4)	40,789	1,532.3 (1414.5 - 1657.3)
Atlantic Canada**	7,401	27.0 (3.4 - 97.7)	7,401	608.0 (443.6 - 813.7)
**Total**	**87,201**	**61.9 (46.5 - 80.8)**	**87,201**	**1,449.5 (1370.7 - 1531.7)**

* Province or region of residence at the time of CBS hepatitis B first-time blood donor screening.

** Atlantic Canada is a summary of CBS first-time donors who were screened for hepatitis B who resided in the provinces of New Brunswick, Nova Scotia, Prince Edward Island, and Newfoundland & Labrador at the time of blood donor screening.

Table S3. Output from univariate logistic regression model of case-control interviews with clinical scenario 2 (likely chronic infection) as the dependent variable.

Variable	Odds Ratio (OR)	95% CI	P Value
Ever received a blood transfusion	2.58	1.53 – 4.37	0.0004
Lived with someone who had hepatitis (any)	10.49	4.94 – 22.13	<0.0001
Lived with someone who had liver disease	2.75	1.00 – 7.56	0.0500
Vaccinated for hepatitis B	0.56	0.41 – 0.76	0.0003
High-risk ethnic origin[1]	7.22	5.38 – 9.77	<0.0001
Body piercing	0.40	0.20 – 0.77	0.0064
10+ sexual partners in lifetime	0.35	0.22 – 0.58	<0.0001
Tattoo	0.36	0.23 – 0.59	<0.0001
Born in high-risk country (>5% prevalence)*	9.21	6.62 -12.81	<0.0001

*Ethnic origin was classified as high-risk if the donor self-reported their ancestral ethnicity to be from a high-risk country (5% or higher HBV prevalence (Schweitzer et al., 2015)); these ethnicities included Asian (unspecified, East, Southeast, West, and South), African, Chinese, and Filipino. People of African, Asian , Chinese, and Filipino ethnic origin have an increased likelihood of having been born in or having lived with people born in HBV-endemic countries and were considered "high-risk"
